# Cancer-associated fibroblasts: challenges and opportunities

**DOI:** 10.18632/oncotarget.28385

**Published:** 2023-03-21

**Authors:** Hossein Tavana, Gary D. Luker

**Keywords:** fibroblasts, cancer-associated fibroblasts, tumor microenvironment, therapeutic targeting

## Cancer-associated fibroblasts (CAFs)

Fibroblasts typically are mesenchymal-lineage cells that produce collagen and other extracellular matrix molecules to provide structural support for normal tissues [1]. As part of normal physiology, damaged tissues produce various signaling cues to activate fibroblasts from a quiescent state to facilitate tissue repair. Activated fibroblasts proliferate; increase contractility to exert force on the extracellular matrix; secrete additional extracellular matrix proteins; and remodel the local matrix environment [2]. Damaged tissues present striking analogies to solid tumors, enabling cancers to co-opt functions of activated fibroblasts. Tumor microenvironments (TME) are abundant in soluble signaling factors, including those that cancer cells secrete to activate resident fibroblasts. These activated fibroblasts are known as CAFs. Unlike in normal physiology where fibroblasts resume a quiescent state following tissue repair, CAFs remain active in tumors due to the continuous exposure to the secretome of cancer cells. In 1986, Dr. Harold F. Dvorak published an essay where he described similarities between events in solid tumors to those in tissue repair and suggested that tumors are like wounds that do not heal [3].

## Origins and characteristics of CAFs

Identifying precise origins of CAFs in human tumors is challenging due to the difficulty with longitudinal sampling of lesions, lineage tracing of cells, and lack of a definitive marker for fibroblasts or CAFs [1]. While studies suggest the majority of CAFs originate from resident fibroblasts, there is also evidence for other origins. For example, two studies showed that bone marrow-derived mesenchymal stromal cells (BM-MSCs) were recruited to primary breast tumors and to lung metastases and differentiated to PDGFRα^−^ CAFs [4]. In the presence of weakly metastatic breast cancer cells, BM-MSCs developed tumor-promoting paracrine CCL5 signaling characteristics of CAFs [5]. In addition, CAFs can originate from adipose stromal cells trafficking into tumors, endothelial-to-mesenchymal transition, and pericytes [6–8]. Mechanistically, CAFs can develop through various signaling events including paracrine TGFβ mediated SMAD signaling [9], autocrine TGFβ and SDF-1 signaling [10], contact with cancer cells and resulting Jagged-1 mediated Notch2 activation [11], inflammatory cytokines IL-1 and IL-6 through NFκB and STAT transcription factors [12], proinflammatory cytokine LIF mediated epigenetic switch that activates JAK1/STAT3 signaling [13], and matrix mediated mechanotransduction [14, 15]. In histological images, CAFs have a distinct morphology and lack expression of markers for epithelial cells, endothelial cells, and leukocytes. CAFs typically are identified by morphology and expression of markers such as platelet-derived growth factor receptor-α or β (PDGFRα or PDGFRβ), vimentin, α-smooth muscle actin (αSMA), and fibroblast activation protein (FAP).

## Heterogeneity of CAFs

Much like cancer cells, CAFs are not a single population but exhibit considerable heterogeneity. A recent integrated analysis of data from single-cell sequencing of head and neck squamous cell carcinoma, lung, and melanoma tumors identified six distinct subtypes of CAFs [16]. Subtypes included myofibroblast-like CAFs with high expression of activated fibroblast marker *ACTA2* and smooth muscle cell markers *MYH11*, *MCAM*, *TAGLN*, and *MYLK*; desmoplastic-like CAFs with enriched expression of collagen and ECM remodeling; two-inflammatory-like CAFs with high expression of *CXCL12* and *CXCL14* in the first subtype and *CXCL2* and *NFκB* signaling pathway in the second subtype; normal-like CAFs enriched for markers of homeostasis; and proliferative-like CAFs with elevated expression of cell cycle markers such as *BIRC5* and *TOP2A*. The resulting subtype-specific gene sets were used to determine the abundance and prognostic values of the CAFs subtypes. The analysis across 31 cancer types from TCGA showed that most CAFs subtypes correlated either strongly or moderately with a poor prognosis or correlated with a favorable prognosis in specific cancers. A similar single-cell RNA-Seq analysis of mesenchymal cells from tumors of a breast cancer mouse model led to four different CAFs subtypes [17].

Despite general associations with a worse overall survival, subsets of CAFs may function as antigen presenting cells that enhance anti-tumor immunity, underscoring heterogeneity and suggesting that CAFs may turn against cancer cells [18]. In addition to their molecular heterogeneity, CAFs may also present spatial heterogeneity in tumors. For example, histological analysis of pancreatic tumors showed that CAFs proximal to cancer cells display a myofibroblast-like contractile phenotype, whereas those distal to cancer cells have an inflammatory-like phenotype with high IL-6 levels [19]. Another puzzling question is whether CAFs subtypes have the plasticity to transition between different cell states, as CAFs from mouse PDAC showed the ability to switch between αSMA^+^ myofibroblast-like and IL-6-producing inflammatory-like subtypes [20]. Overall, identifying which subtypes of CAFs are tumor promoting and cause therapy resistance and which cancer types may benefit from CAFs subtypes-targeted therapies may help improve outcomes for patients.

## Functions of CAFs

Unlike normal fibroblasts in a quiescent state, CAFs are proliferative, migratory, and metabolically active. CAFs in tumors have two broad range of functions: (i) mechanical remodeling of the tumor microenvironment and (ii) soluble signaling with cancer cells. CAFs synthesize and secrete different proteins and matrix-crosslinking enzymes and mechanically remodel and stiffen the extracellular space. Stiff tumors reduce transport of drugs, limit infiltration of immune cells, and lead to hypoxia, promoting tumor angiogenesis and cancer cell survival and proliferation. Tumor stiffness is a poor prognostic factor in breast and other solid cancers [21, 22]. CAFs also secrete various proteases to degrade the extracellular matrix and generate permissive paths for invasive cancer cells. In addition, CAFs produce a broad array of soluble factors to directly interact with cancer cells in different solid tumors. These factors include growth factors (e.g., VEGF, HGF, and TGF-β), chemokines (e.g., CXCL9 and CXCL12), cytokines (e.g., IL-6, IL-8, and IL-10), exosomes, extracellular vesicles, and metabolites. The following provides two examples. CXCL12 secreted by CAFs signals through receptors CXCR4 and/or ACKR3 to drive numerous processes required for tumor growth and metastasis, including proliferation, glycolytic metabolism, and migration/invasion [23–28]. CAFs secrete various isoforms of CXCL12 with CXCL12-γ, an isoform with high binding affinity to heparan sulfate proteoglycan extracellular matrix molecules, producing greatest effects to drive metastasis [29]. CXCL12 also attracts suppressive immune cells, such as myeloid derived suppressor cells and T regulatory cells, to tumor environments [30]. Secretion of HGF by CAFs in both primary and metastatic breast tumor environments drives epithelial-to-mesenchymal transition (EMT), local invasion, and activation of oncogenic signaling pathways promoting proliferation and survival [31]. These data suggest that blocking HGF or signaling through the receptor MET on breast cancer cells could reduce both primary tumor growth and metastasis [32, 33]. Beyond these specific examples, interactions between CAFs and cancer cells mediated by soluble factors generally promote protumorigenic biological processes and disease progression by increasing tumor growth, cancer cell invasion into the tissue stroma to mediate metastasis, tumor angiogenesis, resistance to chemotherapies and targeted therapies, and immune system evasion. CAFs generate immunosuppressive effects by diminishing tumor cytotoxicity of effector CD8^+^ T cells, polarizing macrophages to an immunosuppressive M2 state, and suppressing NK cell activation and cytotoxicity. Extracellular matrix produced by CAFs also may exclude immune cells from a tumor, producing immune-excluded or immune desert tumors that do not respond to current checkpoint immunotherapy antibodies.

## CAFs for detection and treatment of cancer

Considering the abundance and the various roles of CAFs in the TME, many preclinical studies and clinical trials have investigated the utility of imaging CAFs to detect cancer. Imaging agents based on inhibitors for fibroblast activation protein (FAPI), a type II transmembrane serine protease expressed in >90% of epithelial cancers, show tremendous promise to detect various types of cancers [34]. Positron emission tomography (PET) FAPI imaging agents potentially improve detection of some cancers relative to fluorodeoxyglucose (FDG), the current standard for oncology imaging [35].

Beyond detection of cancer, CAFs are being actively pursued as targets for therapy. However, the heterogeneity of CAFs and their ability to potentially assume different phenotypes introduce significant challenges. One approach that has been pursued in preclinical studies is to deplete or normalize CAFs in tumors. In murine PDAC tumors, depleting αSMA^+^ CAFs led to an immunosuppressive TME with undifferentiated and invasive tumors [36], whereas ablating FAP^+^ CAFs allowed immunogenic control of tumor growth [37]. This suggests the importance of identifying and targeting specific subsets of CAFs in solid tumors. Treating PDAC tumor-harboring mice with a ligand for vitamin D receptor shifted stellate cells toward an inactive state with reduced inflammation, fibrosis, and tumor volume, increased intratumor drug availability, and significantly improved survival [38]. Because CAFs secrete soluble signaling factors in the TME, another appealing approach is to disrupt interactions of signaling molecules of CAFs and cancer cells. CXCL12, HGF, VEGF, TGFβ, and IL-6 are such ligands that promote tumor progression across different cancers. There is ample evidence form preclinical studies that blocking CAFs-cancer cell interactions inhibits tumor growth, angiogenesis, invasion, and metastasis. Several clinical trials have also used this strategy, such as blocking CXCL12-CXCR4 signaling in PDAC and blocking TGFβ signaling in breast, lung, PDAC, renal, colorectal, and hepatocarcinoma [39, 40]. This approach may also be combined with another treatment such as anti-PD-1 or chemotherapeutics to increase anti-tumor effects.

Generating an immunosuppressive TME is one of the main functions of CAFs. Hence, there is a great interest in developing immunotherapy approaches against CAFs. For example, an oral DNA vaccine was used to elicit CD8^+^ T cell-mediated cytotoxicity of FAP^+^ CAFs [41]. This strategy suppressed primary tumor growth and metastasis of multidrug-resistant murine colon and breast carcinoma; decreased type I collagen content of the tumors; enhanced the uptake of chemotherapeutics up to 70%; and prolonged survival. Another study showed that the combined use of anti-FAP and CAR T cells enhances anti-tumor immunity in immuno-deficient xenografts [42]. Investigators also are developing FAPI-targeted radiopharmaceuticals with alpha- or beta-emitting radionuclides as theranostic agents for detection and treatment of cancer [43]. Overall, anti-CAF immunotherapeutics combined with chemotherapies is a promising new approach for the treatment of solid cancers and several such studies have entered clinical trials.

## CONCLUSIONS

CAFs are a biologically complex subset of cells in the TME with predominantly protumorigenic and immunosuppressive functions. CAFs present an attractive target for therapies. Delineating cross-talk of CAFs with cancer cells and other stromal cells, uncovering the role of CAFs in resistance to chemotherapies and immunotherapies, addressing challenges associated with CAFs heterogeneity to develop CAFs subtype-targeted therapies in the context of specific tumor types, and addressing the potential toxicity of such therapies especially when combined with other treatments will expedite the ongoing efforts for the translation of therapies against CAFs.
